# Endoplasmic Reticulum Stress Mediates Methamphetamine-Induced Blood–Brain Barrier Damage

**DOI:** 10.3389/fphar.2017.00639

**Published:** 2017-09-14

**Authors:** Xiaojuan Qie, Di Wen, Hongyan Guo, Guanjie Xu, Shuai Liu, Qianchao Shen, Yi Liu, Wenfang Zhang, Bin Cong, Chunling Ma

**Affiliations:** ^1^Hebei Key Laboratory of Forensic Medicine, Department of Forensic Medicine, Hebei Medical University Shijiazhuang, China; ^2^Department of Anesthesiology, The Third Hospital of Hebei Medical University Shijiazhuang, China; ^3^The 8th Brigade of General Division of Criminal Investigation, Beijing Municipal Public Security Bureau Beijing, China

**Keywords:** methamphetamine, blood–brain barrier, brain endothelial cells, endoplasmic reticulum stress, mitochondrial dysfunction, apoptosis

## Abstract

Methamphetamine (METH) abuse causes serious health problems worldwide, and long-term use of METH disrupts the blood–brain barrier (BBB). Herein, we explored the potential mechanism of endoplasmic reticulum (ER) stress in METH-induced BBB endothelial cell damage *in vitro* and the therapeutic potential of endoplasmic reticulum stress inhibitors for METH-induced BBB disruption in C57BL/6J mice. Exposure of immortalized BMVEC (bEnd.3) cells to METH significantly decreased cell viability, induced apoptosis, and diminished the tightness of cell monolayers. METH activated ER stress sensor proteins, including PERK, ATF6, and IRE1, and upregulated the pro-apoptotic protein CHOP. The ER stress inhibitors significantly blocked the upregulation of CHOP. Knockdown of CHOP protected bEnd.3 cells from METH-induced cytotoxicity. Furthermore, METH elevated the production of reactive oxygen species (ROS) and induced the dysfunction of mitochondrial characterized by a Bcl2/Bax ratio decrease, mitochondrial membrane potential collapse, and cytochrome c. ER stress release was partially reversed by ROS inhibition, and cytochrome c release was partially blocked by knockdown of CHOP. Finally, PBA significantly attenuated METH-induced sodium fluorescein (NaFluo) and Evans Blue leakage, as well as tight junction protein loss, in C57BL/6J mice. These data suggest that BBB endothelial cell damage was caused by METH-induced endoplasmic reticulum stress, which further induced mitochondrial dysfunction, and that PBA was an effective treatment for METH-induced BBB disruption.

## Introduction

Methamphetamine (METH) is recognized as a psychostimulant, which is characterized by wide abuse and high addiction (Chomchai and Chomchai, 2015). Long-term use of METH causes damage to neurons and the blood–brain barrier (BBB) can induce mounting of neurodegenerative diseases (Mursaleen and Stamford, 2016). The effects of METH on BBB disruption mainly include hyperpyrexia, glucose transporter impairment, oxidative stress (OS), inflammation and increases in plasma ammonia. The roles of OS underlying BBB disruption have been well-documented; in fact, OS is involved in BBB disruption in numerous neurodegenerative disorders, such as concomitant BBB disruption (Northrop and Yamamoto, 2015).

The endoplasmic reticulum (ER), a vital organelle for protein secretion and modification. Disruption of proper ER function results in ER stress, which triggers the unfolded protein response (UPR) (Kogel et al., 2003; Resende et al., 2008; Alberdi et al., 2013; Pinkaew et al., 2015). During unfolded protein accumulation in sustained ER stress, glucose-related protein (Grp78)/Bip, a major chaperone protein, detaches from the transmembrane ER signaling proteins pancreatic ER elF2a kinase (PERK), activating transcription factor 6 (ATF6) and inositol-requiring enzyme 1 (IRE1). PERK, ATF6, and IRE1 are triggered by the binding of chaperones to misfolded proteins. Excessive and prolonged ER stress ultimately causes apoptosis by promoting the expression of the CCAAT/enhancer-binding protein homologous protein (CHOP) (Shen et al., 2004; Schroder and Kaufman, 2005; Ron and Walter, 2007). It is demonstrated that ER stress plays a key role in the onset and progression of neurodegenerative diseases (Mercado et al., 2016; Thummayot et al., 2016). ER stress was also involved in METH-induced umbilical vein endothelial cell injury (Cai et al., 2016). Our previous studies have found that ER stress is related to restraint stress-induced rat kidney injury and hippocampal damage (Geng et al., 2013; Zhang et al., 2014). Whether ER stress is involved in METH-induced BBB damage has not been determined yet.

Mitochondrial dysfunction affect the pathways of apoptotic cell death and is characterized by decreased mitochondrial membrane potential (MMP) and increased protein expression of Bcl-2 family and promoted the release of cytochrome c (Jiang et al., 2017). While loss of MMP has been reported as an early event in some apoptotic processes (Ismail et al., 2013), cytochrome c release has been considered to be a major effector of apoptosis (Ben Safta et al., 2015). Prolonged ER stress could accelerate OS (Choi et al., 2010); in turn, OS has been known to accelerate ER stress and activate apoptotic signaling pathways (Tsujii et al., 2015; Go et al., 2016). The interplay between oxidative and ER stress has gradually gained attention. For example, it has been reported that CHOP affects the generation of reactive oxygen species (ROS) in neurons (Kim et al., 2013). This interplay has also been found in thiamine deficiency and neurodegeneration, where OS disrupts the ER redox state and thereby disrupts proper protein folding in the ER (Liu et al., 2016). Wang et al. (2016) reported that misfolded proteins in the ER could induce the production of ROS. However, there are no reports of any such interplay in METH-induced BBB disruption as of yet.

This study aimed to elucidate the mechanism of METH-induced BBB disruption through investigating the role of ER stress and the potential oxidative/ER stress interplay. Considering that one component of BBB is brain microvascular endothelial cells (BMVECs), we used immortalized mouse BMVECs (bEnd.3) to model BBB damage *in vitro*. Moreover, we used C57BL/6J mice to analyze the roles of ER stress in METH-induced BBB disruptions *in vivo*.

## Materials and Methods

### Drugs and Reagents

DL-methamphetamine (purity > 95%, supplied by Public Security Bureau of Beijing Municipality) was dissolved in a 0.9% saline solution. The ER stress inhibitors salubrinal (SB, SML-0951) and sodium 4-phenylbutyric acid (PBA, ab141253) were from Sigma (United States) and Abcam (Cambridge, MA, United States), respectively. The NADPH oxidase inhibitor apocynin (ab120615) and ROS scavenger *N*-tert-butyl-α-phenylnitrone (NBP, RQ75L-QN) were obtained from Abcam and Tokyo Chemical Industry (Tokyo, Japan), respectively. Antibodies were obtained from the following sources: CD31 (ab28364), claudin5 (ab15106), Bip (ab21685), p-IRE1 (ab48187), IRE1 (ab37073), CHOP (ab11419), and cytochrome c (ab13575) were from Abcam; occludin (13409-1-AP), bcl-2 (12789-1-AP), and bax (50599-2-1g) were from Proteintech (Chicago, IL, United States); ATF6 (BS6476), GAPDH (AP0066), and COX IV (BS2186) were from Bioworld (St. Louis, MO, United States); p-PERK (sc-32577) and PERK (sc-9477) were from Santa Cruz (Dallas, TX, United States).

### Cell Culture and Stimulation

The bEnd.3 cells (Wan et al., 2014; Hun Lee et al., 2015) were obtained from ATCC and cultured in ECM (ScienCell, San Diego, CA, United States), which contained 10% fetal bovine serum (Gibco, Carlsbad, CA, United States) and 1% penicillin/streptomycin (Invitrogen, Carlsbad, CA, United States) under a humidified cell culture incubator at 37°C and with 5% (v/v) CO_2_. The bEnd.3 cells were then exposed to 250 μM, 500 μM, and 1 mM METH for 24, 48, and 72 h, and prepared for the following experiments (Fernandes et al., 2015).

### Animals

Three-month-old male C57BL/6J mice, weighing 18–25 g, were obtained from Beijing Vital River Laboratory Animal Technology Co. Ltd., (Beijing, China). All experiments were performed according to the Guideline for the Care and Use of Laboratory Animals. The experiments were approved by the Local Committee on Animal Care, Use and Protection of Hebei Medical University. Mice were randomly grouped into four experimental groups: Saline, METH, METH+PBA, and PBA. METH (5 mg/kg body weight, 3-h intervals) was injected intraperitoneally four times a day for 1 day. PBA (50 mg/kg body weight) was intraperitoneally injected 30 min before METH injection. One day after the first injection, evaluation of BBB permeability was performed and the animals were killed for tissue collection.

### Immunofluorescence

The bEnd.3 cells were seeded on coverslips and fixed in 4% paraformaldehyde for 20 min at 25°C. After blocking solution was treated, the cells were incubated with primary antibodies (anti-CD31 1:50, anti-cytochrome c 1:200) overnight at 4°C. Cells were clearly washed with PBS and incubated with the second antibody for 40 min at 37°C. After washing in PBS for 30 min; nuclear staining was performed using 4′,6-diamidino-2-phenylindole (DAPI). Cells were detected by an SP8 Leica confocal microscope (Leica Biosystems, Wetzlar, Germany).

### Methyl-Thiazolyl-Tetrazolium (MTT) Assay

The measurement of cell viability was carried out by MTT assay. Briefly, bEnd.3 cells were seeded at a density of 1 × 10^4^ cells per well. Then the cells were treated with METH. A volume of 10 μl MTT (5 mg/ml) was added to each well. After an incubation for 4 h, 150 μl of 10% SDS were added and treated for 7–17 h at 37°C. Absorbance was measured at OD = 570 nm by a full-length microplate reader (Thermo Fisher, Waltham, MA, United States).

### Flow Cytometry

Flow cytometry was carried out to measure cell apoptosis, which was described previously (Yin et al., 2015). The cells were harvested and washed twice with PBS. Thereafter, cells were labeled with annexin V-FITC and propidium iodide (PI) (BD Biosciences). Fluorescent signals were measured with a flow cytometer (FACSCalibur, BD Biosciences).

### TUNEL Assay

To determine relative levels of cell apoptosis, the S7165 ApopTag^®^Red *In Situ* Apoptosis Detection Kit (Millipore, Billerica, MA, United States) was used. Briefly, the cells were seeded on slides. After permeabilizing and fixing for a second time, the cells were equilibrated and labeled. Then, DAPI was used to visualize cell nuclei. Cells were detected by an SP2 Leica confocal microscope. The numbers of TUNEL-positive cells were counted in five randomly selected fields (400× magnification) and the percentages calculated against total number of DAPI-stained cells. Two independent observers who were blinded to the experimental conditions performed the counts and calculated the average number of TUNEL-positive cells. Data were collected from more than three independent experiments performed in triplicate.

### Transepithelial Electrical Resistance (TEER)

Transepithelial electrical resistance was used to determine the integrity of brain endothelial monolayers using the Millicell Voltohmmeter (Millipore) with STX01 chopstick electrodes. In brief, bEnd.3 cells were inoculated in transwell inserts of 24-well plates at a density of 5 × 10^4^/well. Prior to use, the machine was calibrated, then the longer electrode was placed so as to touch the bottom of the dish while the shorter electrode was prevented from reaching the bottom of the insert. The readings were corrected by transwell inserts with no cells (subtracted from each experimental measurement), then divided by filter size.

### Western Blot Analysis

Protein extracts of bEnd.3 cells and brain tissue were loaded on 8–15% polyacrylamide minigels (Bio-Rad Laboratories, Hercules, CA, United States), and proteins were separated using SDS-PAGE. Further, protein was electroblotted to PVDF membranes and blocked using 5% non-fat dry milk at 37°C for 1 h. The membranes were incubated with 5% non-fat dry milk containing the appropriate primary antibody overnight at 4°C. After primary incubation, blots were incubated in the secondary antibody for 1 h at room temperature. An imager (LI-COR, Lincoln, NE, United States) was employed to detect the emitted light of the blot.

### Real-Time PCR Arrays

Total RNA was derived from bEnd.3 cells with an RNeasy Mini Kit (SABiosciences, Qiagen), and 1 μg of RNA was taken out for reverse-transcription using the RT^2^ First Strand Kit (SABiosciences, Qiagen). Expression of 84 UPR pathway-related genes in METH-treated cells was compared to controls using RT^2^ Profiler^TM^ PCR Array Mouse UPR (PAMM-089A). Heat maps and scattered plots were created with the SA Biosciences RT^2^ Profiler PCR Array Data Analysis Web Portal. If the fold change was greater than 2 and *p* < 0.05, the genes were considered significantly different across conditions and described in **Figure 2**.

### Cell Transfection

Short interfering RNA (siRNA) targeting CHOP (si-CHOP) (GGA AGC AAC GCA TGA AGGA) was obtained from Ribo Life Science Co. (Suzhou, Jiangsu province, China). Transfection of siRNA into bEnd.3 cells was conducted. Briefly, bEnd.3 cells were incubated at 60% confluence in 24-well plates. We used the protocol that included the Lipofectamine^®^2000 transfection reagent (Invitrogen), in which 20 pmol si-CHOP or scrambled siRNA was added to 50 μL of serum-free medium. In another tube, 1 μL of Lipofectamine^®^2000 was added to 50 μL of ECM medium and incubated for 5 min at 25°C. Both tubes were mixed and incubated for 20 min at 25°C. Thereafter, the cells were incubated with 100 μL of this mixture.

### Measurement of ROS

To determine the level of ROS, the oxidative conversion of 2,7-dichlorofluorescein diacetate (DCFH-DA) to the fluorescent compound dichlorofluorescein (DCF) was measured. Cells were cultured in 24-well plates at 5 × 10^4^ cells per well or in 96-well plates at 1 × 10^4^ cells per well. The medium was treated with aspiration and replaced by serum-free medium. DCFH-DA (Beyotime Company, Shanghai, China) stock solution (10 mM) was diluted to a final concentration of 10 μM using serum-free medium (V/V = 1:999), and then added to the plates for 30 min. DCF fluorescence was measured at 488-nm excitation and 520-nm emission using an SP8 Leica confocal microscope or a microplate reader (Infinite 200, Tecan, Mannedorf, Switzerland).

### MMP Analysis

The change of MMP in the bEnd.3 cells was monitored with the MMP Detection Kit with JC-1 (Beyotime Company). Briefly, bEnd.3 cells were seeded in 6-well plates with 2.5 × 10^5^ cells per well. After drug treatments, the cells were harvested and treated with a JC-1 working solution (5 μg/mL) for 20 min at 37°C in a 5% CO_2_ incubator. After washing in cold JC-1 staining buffer, the fluorescence intensity of the cells was monitored at excitation/emission wavelengths of 514/529 nm (JC-1 monomers) and 585/590 nm (JC-1 aggregates) using a microplate reader (Infinite 200, Tecan, Mannedorf, Switzerland). The MMP of bEnd.3 cells was calculated as the fluorescence ratio (JC-1 aggregates: monomers).

### Preparation of Cytosolic and Mitochondrial Fractions

Collecting the cells with different treatments and mitochondria of cells was isolated with a Cell Mitochondria Isolation Kit (Beyotime Company). Suspending the cells with 1 mM of phenylmethanesulfonyl fluoride in mitochondrial isolation buffer, after that the cells were homogenized and then centrifuged at 4°C for 10 min, and the resulting supernatant was subjected to centrifugations with the speed of 11,000 × *g* for another 10 min to separate the cytosolic (supernatant) from the mitochondrial fractions (pellets).

### Evaluation of BBB Permeability

Evans Blue and sodium-fluorescein (NaFluo) were used to evaluate large and small solute permeability of the BBB, respectively. The procedures were performed in accordance with previous methods (Ramirez et al., 2009; Martins et al., 2011). Briefly, mice were injected intraperitoneally with 2% NaFluo (2.5 ml/kg, Sigma) or into the tail vein with 2% Evans Blue (4 ml/kg, Sigma). The tracers were circulated for 30 min and 2 h, respectively. The mice were then transcardially perfused with saline until colorless perfusate was observed. The animals were then sacrificed under euthanasia and the brains were immediately collected. Tissue was homogenized in 3 ml of 50% trichloroacetic acid. The homogenate was centrifuged at 25°C for 10 min, 5 M NaOH was used to neutralize the supernatant. Measurements of NaFluo and Evans Blue fluorescence were measured at the wavelengths of excitation/emission at 440/525 nm and 550/620 nm, respectively, by a microplate reader (Infinite 200, Tecan, Mannedorf, Switzerland).

### Data Analysis

The data are presented as the means ± SD. One-way ANOVA and Student’s *t*-test were performed for statistical analysis as appropriate. A *p*-value < 0.05 indicated statistical significance. All analysis were conducted using SPSS 16.0 (Chicago, IL, United States).

## Results

### METH-Induced bEnd.3 Cell Dysfunction

**FIGURE 1:**
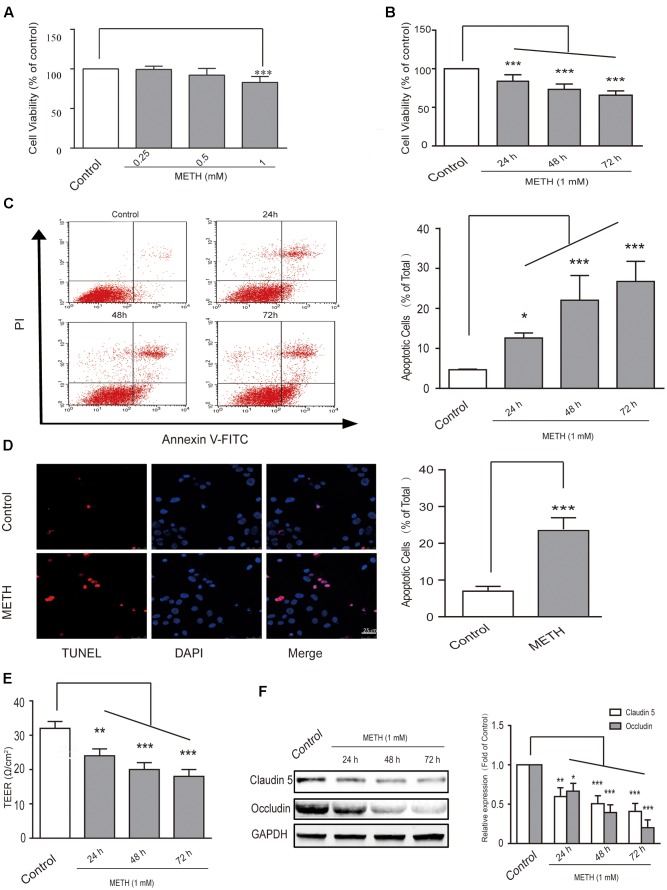
Methamphetamine (METH) induction of brain endothelial cell dysfunction *in vitro*. **(A)** Cell viability was assessed using the MTT assay after bEnd.3 cells were exposed to 0.25, 0.5, and 1 mM of METH for 24 h, and 1 mM of METH significantly inhibited bEnd.3 cell viability. **(B)** Cell viability was assessed using the MTT assay after bEnd.3 cells were exposed to 1 mM of METH for 24, 48, and 72 h, and METH induced a significant, time-dependent decrease in cell viability. **(C)** Apoptosis was measured by flow cytometry in bEnd.3 cells treated with 1 mM of METH for 24, 48, and 72 h, which significantly increased cell apoptosis. **(D)** Apoptotic cells were demonstrated as shown by TUNEL-positive cells (red). The nucleus was stained with DAPI (blue) in control (upper panels, control) and METH (lower panels, METH) treated conditions, and METH induced a significant increase in cell apoptosis. **(E)** TEER was measured when bEnd.3 cells were treated with 1mM of METH for 24, 48, and 72 h; 1 mM of METH reduced TEER after 24 h, and the reduction was gradually enhanced after 48 and 72 h. **(F)** Western blot analysis of tight junction proteins claudin5 and occludin at different time points of 24, 48, and 72 h after treatment with METH. ^∗^*p* < 0.05, ^∗∗^*p* < 0.01, ^∗∗∗^*p* < 0.001 compared with the control. Data are expressed as the mean ± SD of three separate experiments performed in duplicate (*n* = 3).

First, in order to select the appropriate concentration of METH for the subsequent experiments, we used 250 μM, 500 μM, and 1 mM of METH to treat bEnd.3 cells; then, MTT assay was performed to observe cell viability. The results showed that 250 and 500 μM of METH had no significant influence on bEnd.3 cell viability, but 1 mM of METH significantly inhibited bEnd.3 cell viability (**Figure 1A**). Thus, we chose 1 mM of METH for subsequent study. Next, we detected cell viability using the MTT assay after bEnd.3 cells were exposed to METH (1 mM) for 24, 48, and 72 h. Exposure of cells to METH showed a significantly decrease of cell viability in a time-dependent manner (**Figure 1B**). Flow cytometry and TUNEL assays were used to detect apoptosis of bEnd.3 cells after METH treatment. As shown in **Figure 1C**, the apoptotic ratio significantly and gradually increased after METH treatment for 24, 48, and 72 h. Moreover, the TUNEL assay showed that compared with the control group (6.97 ± 1.31%), 1 mM METH caused a significant increase in apoptosis rate at 24 h (22.31 ± 2.50%, *p* < 0.001) (**Figure 1D**). These results suggest that METH could reduce cell viability and induce the apoptosis of bEnd.3 cells.

Transepithelial electrical resistance measurement supplies an efficient and easy evaluation for assessing of BBB integrity using an *in vitro* model. In our study, we found that 1 mM of METH reduced TEER after 24 h, and that the reduction was gradually enhanced after 48 and 72 h of METH treatment (**Figure 1E**). Furthermore, our results revealed that METH treatment gradually reduced the expression of tight junction (TJ) proteins claudin5 and occludin (**Figure 1F**). The results suggest that the integrity of BBB endothelial bEnd.3 cells could be destroyed by METH, partly via down-regulation of tight junction proteins. In summary, METH treatment reduced cell viability through induction of apoptosis and appeared to induce damage to the bEnd.3 cells that would diminish the tightness (and thus the strength) of the BBB.

### METH-Induced Prolonged ER Stress in bEnd.3 Cells

**FIGURE 2:**
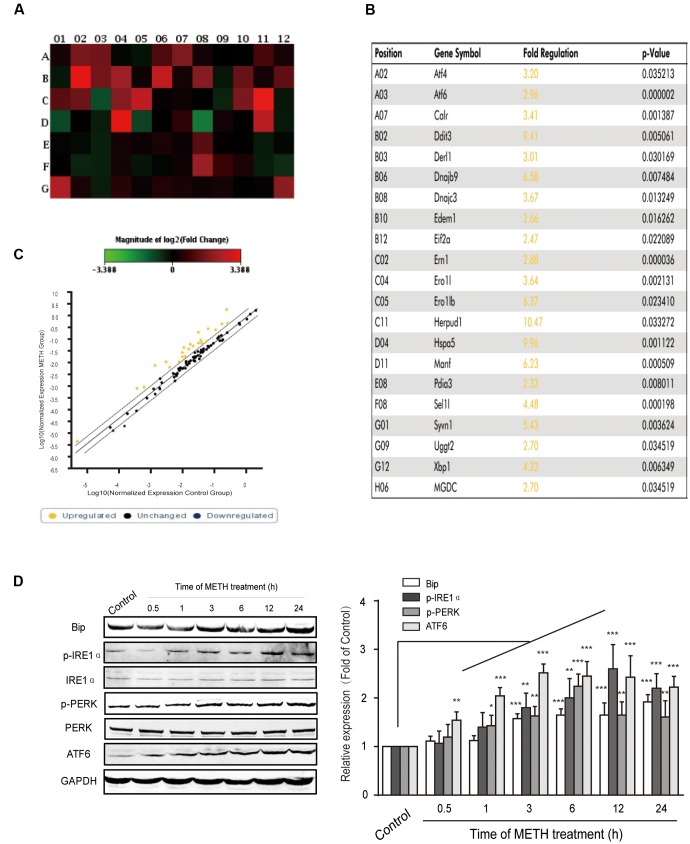
Time-dependent effects of methamphetamine on sensors and chaperones of ER stress. The bEnd.3 cells were incubated with 1 mM of METH for 24 h, and the expression of the UPR pathway genes (84 genes) was examined using a PCR array. **(A)** Changes are shown as a hot map, where red indicates upregulation and green indicates downregulation. **(B)** Changes in genes that are greater than two folds or *p* < 0.05 are listed in the table. **(C)** Changes are shown in scattered plot. **(D)** Western blot analysis of lysate at different time points after 1 mM of METH treatment indicates that the increase of p-PERK, p-IRE1α, ATF6, and GRP78/Bip were time-dependent. ^∗^*p* < 0.05, ^∗∗^*p* < 0.01, ^∗∗∗^*p* < 0.001 compared with the control. Data are expressed as the mean ± SD of three separate experiments performed in duplicate (*n* = 3).

It is widely accepted that long time ER stress promotes cell apoptosis. To evaluate whether bEnd.3 cells exposure of METH led to ER stress induction, we identified the expression of numerous critical genes (84 genes) of the UPR pathway using PCR arrays. Our results showed that ER stress affects the expression of many critical genes, such as ATF4, ATF6, Ddit3, Eif2a, Hspa5, and Xbp1, was increased > two-fold after METH treatment for 24 h (**Figures 2A–C**). Further, we selected several typical proteins, including Bip/GRP78, p-IRE1, p-PERK, and ATF6, in the UPR pathway to verify the results of RT-PCR arrays and further determine whether the ER stress was sustained or transient. Briefly, bEnd.3 cells were exposed to METH (1 mM) for 0.5, 1, 3, 6, 12, and 24 h, after which the expression of ER stress related-proteins was assessed. As is presented in **Figure 2D**, exposure of bEnd.3 cells to METH significantly promoted the expression of ER stress sensor proteins in a time-dependent manner, such as p-IRE1, p-PERK, and ATF6. Therefore, these results suggest METH-induced prolonged ER stress in bEnd.3 cells.

### The Downstream Effector CHOP Is Critical to METH-Induced bEnd.3 Cell Dysfunction

**FIGURE 3:**
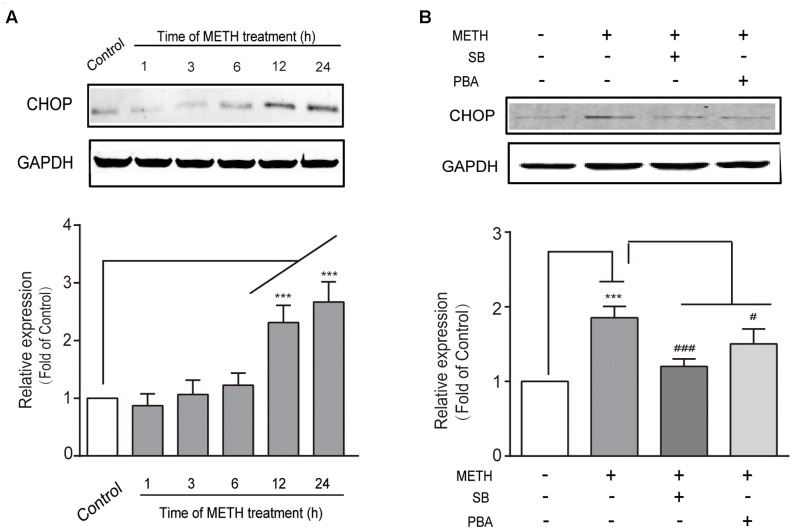
Methamphetamine induced the pro-apoptotic protein CHOP involving ER stress. **(A)** Western blot analysis of lysate at different time points after 1 mM of METH treatment indicates that the increase of CHOP was time-dependent. **(B)** Western blot analysis of lysate indicates that the increase of CHOP was partly reversed by ER stress inhibitors SB or PBA. ^∗∗∗^*p* < 0.001 compared with the control. ^#^*p* < 0.05, ^###^*p* < 0.001 compared with METH-treated cells. Data are expressed as the mean ± SD of three separate experiments performed in duplicate (*n* = 3).

We determined whether the downstream pro-apoptotic pathway effector CHOP was activated following METH treatment. It has reported that CHOP/GADD153 belongs to the bZip transcriptional factors family and is activated by p-IRE1, ATF6, and p-PERK pathways. We found that METH exposure caused a time-dependent increase in CHOP (**Figure 3A**), and that this increase was blocked by pre-treatment of the ER stress inhibitors SB (50 μM) or PBA (5 mM) 30 min before METH treatment (**Figure 3B**). These results suggest that METH induced downstream pro-apoptotic pathways through sustaining ER stress.

**FIGURE 4:**
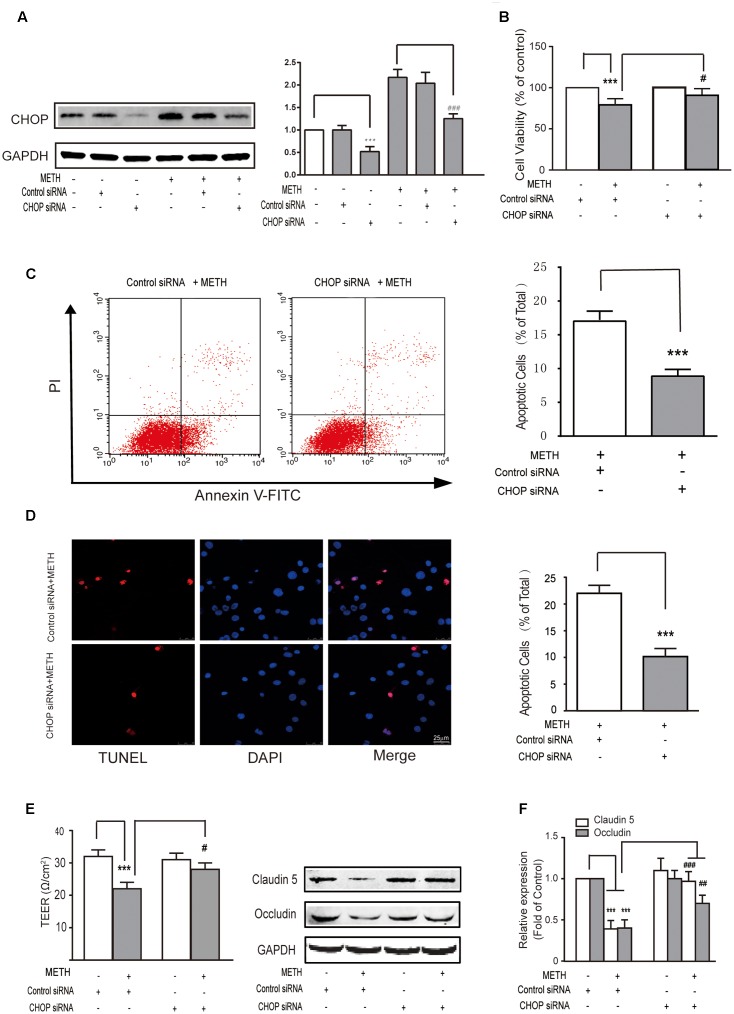
Methamphetamine induced brain endothelial cell dysfunction through prolonged ER stress and downstream pro-apoptotic pathways. After transfection with the control or CHOP siRNA for 24 h, bEnd.3 cells were cultivated with or without METH for 24 h. **(A)** Western blot analysis of the CHOP expression. **(B)** MTT assay for cell viability. **(C)** Apoptotic ratio measured by flow cytometry in METH treated bEnd.3 cells when transfected with siRNA control or siRNA CHOP. **(D)** Apoptotic cells ratio measured by TUNEL assay. **(E)** TEER of cells transfected with siRNA control or siRNA CHOP were compared. **(F)** Western blot analysis of the TJ proteins, caludin5 and occludin. ^∗∗∗^*P* < 0.001 compared with cells treated with siRNA control or cells treated with siRNA control and METH. ^#^*P* < 0.05, ^##^*P* < 0.01, ^###^*P* < 0.001 compared with cells treated with siRNA control and METH. Data are expressed as the mean ± SD of three separate experiments performed in duplicate (*n* = 3).

Subsequently, we further examined the role of CHOP in METH-induced endothelial cell dysfunction using si-CHOP to knockdown the expression of CHOP. The efficiency of si-CHOP was 55% in naïve cells. The transfection of si-CHOP was found to attenuate the level of CHOP increased by METH treatment (**Figure 4A**). Furthermore, si-CHOP could partially restore the reduction of cell viability after METH treatment (**Figure 4B**). The results of these experiments show that the knockdown of CHOP significantly inhibited METH-induced apoptosis (**Figures 4C,D**) and alleviated the disruption of tightness of bEnd.3 monolayers (**Figures 4E,F**). In summary, as the downstream effector of pro-apoptotic pathways, CHOP was involved in ER stress and played a key role in METH-induced bEnd.3 cell dysfunction.

### METH-Induced Generation of ROS and Mitochondrial Dysfunction

**FIGURE 5:**
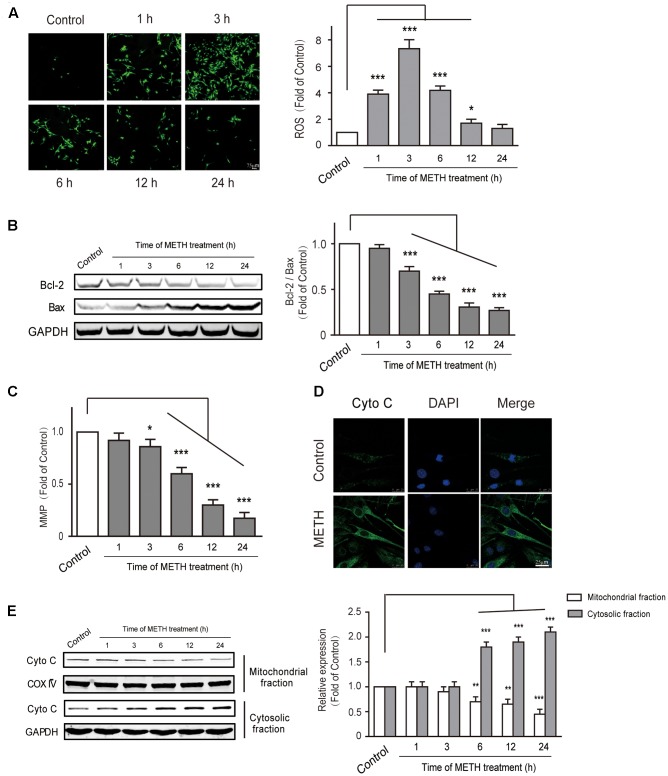
Methamphetamine induced generation of ROS and mitochondrial dysfunction. **(A)** DCFDA assay used for ROS generation. **(B)** Western blot analysis shows that the decrease of Bcl2/Bax was time-dependent. **(C)** METH-induced mitochondrial membrane potential collapse. **(D)** Immunofluorescence shows the release of cytochrome c from the mitochondria to the cytoplasm. **(E)** Western blot analysis of the mitochondrial fraction and the cytosolic fraction of cytochrome c. ^∗^*p* < 0.05, ^∗∗^*p* < 0.01, ^∗∗∗^*p* < 0.001 compared with the control. Data are expressed as the mean ± SD of three separate experiments performed in duplicate (*n* = 3).

Endoplasmic reticulum stress is triggered under oxidative conditions. ROS generation accelerates the production of misfolded proteins, and ultimately results in the ER stress (Tsai et al., 2012; Tsukiyama-Kohara, 2012). We thus aimed to determine whether METH subject to bEnd.3 cells could cause the production of ROS. DCF fluorescence assay was performed to assess the levels of intracellular ROS after bEnd.3 cells were treated with METH (1 mM). We found that METH treatment dramatically promoted the levels of ROS, and it was peaked at 3 h. The ROS levels decreased quickly, and then returned to normalcy after 24 h of treatment, indicating that the increase of ROS levels was rapid and transient (**Figure 5A**). To further investigate the involvement of mitochondrial dysfunction in METH-induced apoptosis of bEnd.3 cells, the Bcl2/Bax, MMP depolarization, and cytochrome c release were detected. As expected, cells supplemented with METH significantly decreased the ratio of Bcl2/Bax and the expression of MMP in a time-dependent manner (**Figures 5B,C**). Western blot analysis and immunofluorescence confirmed that treatment with METH prominently affected the release of cytochrome c, which led to a increase in cytosolic fraction, but a decrease in mitochondrial fraction (**Figures 5D,E**). Together, the data suggest that METH could induce the generation of ROS as well as mitochondrial dysfunction.

### METH-Mediated Induction of ER Stress Was Activated by ROS and Accelerated the Damage to Cells

**FIGURE 6:**
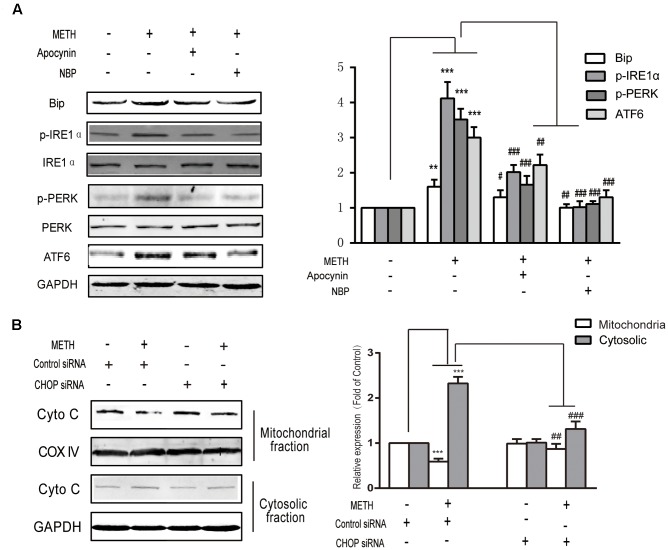
Methamphetamine-mediated induction of ER stress is activated by ROS and accelerates the damage to cells by the release of cytochrome c from the mitochondria to the cytoplasm. **(A)** Markers of ER stress signals were detected by Western blot analysis after treatment with an ROS inhibitor and a scavenger. **(B)** Cytochrome c release after knockdown of CHOP. ^∗∗^*p* < 0.01, ^∗∗∗^*p* < 0.001 compared with the control; ^#^*p* < 0.05, ^##^*p* < 0.01, ^###^*p* < 0.001 compared with METH-treated cells. Data are expressed as the mean ± SD of three separate experiments performed in duplicate (*n* = 3).

To explore the interaction between ROS generation and ER stress response, ROS inhibitors apocynin (a NADPH oxidase inhibitor, 200 μM) or NBP (a ROS scavenger, 100 μM) were subjected to bEnd.3 cells for 30 min before METH treatment. As is presented in **Figure 6A**, both inhibitors prominently ameliorated the METH-induced expression of p-PERK, ATF6, Bip and p-IRE1α. The results indicated the important role of ROS in the METH-mediated ER stress in bEnd.3 cells, and also indicate that the dysregulated expression of p-PERK, ATF6, Bip and p-IRE1α protein was mediated by METH-induced ER stress. Furthermore, CHOP silence inhibited cytochrome c release following METH treatment, thereby implicating CHOP in the METH-induced ER stress response induces the release of cytochrome c, which can activate the caspase family and accelerate cell apoptosis (**Figure 6B**).

### PBA, an ER Stress Inhibitor, Reversed METH-Induced Disruption of the BBB *In Vivo*

**FIGURE 7:**
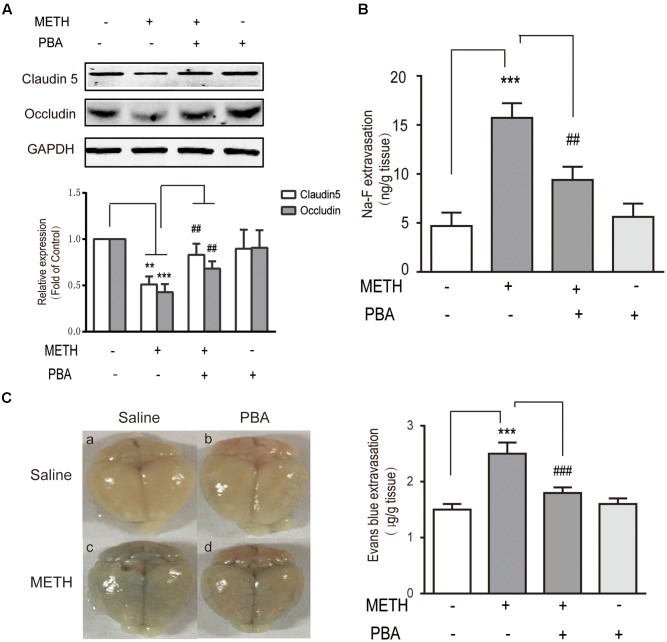
4-phenylbutyricacid (PBA), an endoplasmic reticulum stress inhibitor, partly reversed methamphetamine (METH)-induced disruption of the BBB *in vivo*. Mice were randomly divided into four experimental groups: saline, METH, METH+PBA, and PBA. **(A)** Western blot analysis of claudin5 and occludin. **(B)** The leakage of NaFluo. **(C)** The leakage of Evans Blue: Evans Blue extravasation in a whole brain and quantification. ^∗∗^*p* < 0.01, ^∗∗∗^*p* < 0.001 compared with the control; ^##^*p* < 0.01, ^###^*p* < 0.001 compared with METH-injected mice. Data are expressed as the mean ± SD (*n* = 3).

To further identify whether PBA could reverse METH-induced BBB disruption, we used c57BL/6J mice and intraperitoneally injected METH (5 mg/kg body weight) four times/day for 1 day to generate an acute model of METH abuse (Gou et al., 2015). One group of mice was injected intraperitoneally with PBA (50 mg/kg) 30 min before METH injection, which is often used as an ER stress inhibitor *in vivo* (Jangra et al., 2016). Western blots of TJ proteins to analyze the BBB structure were used, as well as NaFluo (small molecular weight) and Evans Blue (large molecular weight) leakage to test BBB integrity. As illustrated in **Figure 7A**, the administration of METH decreased the expression of TJ proteins, including occludin and claudin5. The decrease of TJ proteins was partly reversed by PBA. Similarly, the permeability to both NaFluo and Evans Blue was also increased following METH exposure, a phenomenon which was partially inhibited by PBA (**Figures 7B,C**). Together, our results suggest that PBA could be a potential drug to reverse METH-induced disruption of BBB *in vivo*.

## Discussion

Methamphetamine can cause BBB disruption, which is proposed to be one mechanism of METH-induced neurotoxicity (Northrop and Yamamoto, 2015). Up until now, the mechanisms responsible for METH-induced BBB disruption have remained largely unknown. The BBB comprises a layer of tightly connected brain microvascular endothelial cells (BMVECs) that interact with other brain cells, including astrocytes, neurons, and pericytes (Tajes et al., 2014). The BMVECs are the main component of the BBB (Tenreiro et al., 2016). The bEnd.3 cells have already been well accepted as an *in vitro* model for BBB (Watanabe et al., 2013). The present study indicated that METH could promote apoptosis and disrupt tight junctions of bEnd.3 cells by inducing ER stress and OS. METH-induced ER stress was activated by ROS and accelerated the damage to cells by cytochrome c release. Furthermore, we illustrated that PBA, an ER stress inhibitor, could partly reverse METH-induced BBB disruption.

Firstly, the dose-effect relationship of METH on cell viability was performed. It was found that the cellular responses to METH were different in several cell types. Low concentration of METH induced cytotoxicity in primary rat brain microvascular endothelial cells (Martins et al., 2013) and human umbilical vein endothelial cells (Ma et al., 2014). While, METH induced cytotoxicity in bovine brain microvessel endothelial cells (Rosas-Hernandez et al., 2016) and bEnd.5 cells (Fisher et al., 2015) only at a high level of METH concentration. Herein, 1 mM of METH was selected for subsequent experiments. Fernandes et al. (2015, 2016) used the same concentration of METH to treat bEnd.3 cells. In our experiment, 1 mM METH-induced toxicity *in vitro* is probably due to the basic properties of METH.

Furthermore, our data have demonstrated that METH inhibited the viability of bEnd.3 cells and subsequently induced apoptosis in a time-dependent manner, illustrating that METH is cytotoxic to bEnd.3 cells. Special characteristics of BMVECs include the presence of tight junctions. Structurally, the inter-endothelial tight junction complexes comprising the membrane proteins occludin and claudins and membrane-directed scaffolding proteins [such as zonula occludens-1 (ZO-1)] contribute to the physical barrier nature of BBB and strictly limit the molecular/cellular influx from circulation (Engelhardt et al., 2014). The down-regulation, fragmentation, or re-distribution of major TJ proteins can result in reduced endothelial barrier tightness (decreased TEER) (Baeten and Akassoglou, 2011). We evaluated the TJ proteins caudin-5 and occludin, which we believe are the most relevant to METH-induced physiopathologic alteration. As a result, we found that METH reduced the expression of claudin-5 and occludin, causing TEER decrease.

Activation of ER stress, which is related to the pathogenesis of various diseases, such as neurodegenerative disorders (Ozcan and Tabas, 2012), type 2 diabetes (Restaino et al., 2017), atherosclerosis (Tufanli et al., 2017), liver disease (Bai et al., 2016), and cancer (Bai et al., 2016). Up until now, studies about the METH-induced involvement of ER stress in the brain have been performed in neuronal cells (Wongprayoon and Govitrapong, 2016), astrocytes (Shah and Kumar, 2016), and C6 cells of glioma cell lines (Tungkum et al., 2017) *in vitro. In vivo* studies were conducted in the midbrain (Takeichi et al., 2012), parietal cortex (Thomas et al., 2010), striatum (Thomas et al., 2010; Beauvais et al., 2011), and ventral tegmental area (Hayashi et al., 2010) of animals, which were often difficult to distinguish for various cell types. Recently, Thomas et al. (2010) demonstrated that neurotoxic amphetamine exposure could cause hyperpyrexia, which leads to the ER stress response in the meningeal vasculature, striatum, and parietal cortex. Furthermore, METH led to type-1 programmed cell death in astrocytes was associated with ATF6, IRE1α, and PERK pathways, which are mediated by ER stress (Shah and Kumar, 2016). Similarly, METH has also been reported to result in apoptotic cell death in SH-SY5Y neuronal cells through the induction of the CHOP protein and spliced X-box binding protein 1, (Wongprayoon and Govitrapong, 2016). In line with this research, we found that ER stress was mediated in the mechanism of METH-induced apoptosis in the brain’s microvascular endothelial cells. Furthermore, cocaine, another addictive drug similar to METH, was reported to regulate ER stress-autophagy involving cocaine-mediated microglia activation (Guo et al., 2015), providing new insights for further studies of METH-related neuroinflammation disease. In addition, we found that the induction of BIP, PERK, p-IRE, and aATF6 was sustained and occurred earlier than the induction of CHOP, suggesting that ER stress may have induced the upregulation of CHOP. Therefore, we used ER stress inhibitors and observed a significant decrease of METH-induced CHOP expression. All major ER stress pathways mediated by IRE1, PERK and ATF6 converged on one transcription factor, CHOP. CHOP is a major mediator of ER stress and an executor of apoptosis. CHOP siRNA treatment reduced astrocyte apoptosis in the rat model of METH (Shah and Kumar, 2016). Previous studies revealed that CHOP mediated apoptosis after cerebral ischemia (Tajiri et al., 2004). Han et al. (2017) showed that cardiomyocytes isolated from CHOP-/- mice were resistant to ER stress-inducing agents. Our data found that the transfection of siRNA CHOP could reverse METH-induced apoptosis, which is concurrent with the results of the previous studies mentioned above. Interestingly, it was also found that CHOP, a major mediator of ER stress, inhibited downregulation of TJ proteins and decreased TEER, a finding supported by published reports that the enhancement of CHOP was accompanied by the disruption of barrier function in retinal pigment epithelial cells (Huang et al., 2015).

It is widely accepted that OS acted as a key character in BBB disruption. Ramirez et al. (2009) reported that METH could disrupt BBB function by inducing OS in primary human BMVECs. The pro-apoptotic protein/inhibitor of apoptosis protein (IAP) modulated the integrity of mitochondrial membranes, which regulate the mitochondrial apoptotic pathways. It has been demonstrated that the decrease of Bcl-2/Bax could disrupt the potential of mitochondrial membranes, lead to the release of cytochrome c, trigger a caspase cascade, and finally result in cell apoptosis (Yuan et al., 2014). Our data demonstrated that METH could significantly decrease the Bcl2/Bax ratio and mitochondrial membrane depolarization, as well as increase cytochrome c release in cytosolic fraction.

The interplay of OS and ER stress has been demonstrated by Liu et al. (2016) who determined that the interaction between OS and ER stress was prevalent in thiamine deficiency and neurodegeneration; in such conditions, OS could disturb the ER redox state, and thereby disrupt proper protein folding in the endoplasmic reticulum. Wang et al. (2016) reported that misfolded proteins in the ER could induce the production of ROS. Jayanthi et al. (2004) reported that METH induced neuronal apoptosis via interaction between endoplasmic reticulum and mitochondria-dependent death cascades, which interacted with the downstream of caspase signaling pathway. We identified that METH-induced ROS induction was rapid and transient, and that ROS inhibitors/scavengers could partly reverse the induction of ER stress-related signals, such as BIP, PERK, IRE, and ATF6, suggesting that ROS could be the initiator of ER stress. Additionally, our data showed that the knockdown of CHOP partly suppressed METH-induced cytochrome c release. These findings demonstrated that METH induced the interplay of ER stress and OS through the ROS/ER stress/cytochrome c release pathway in bEnd.3 cells. Our data are partly consistent with the previous literature.

4-phenylbutyric acid, a ER stress inhibitor, impedes protein misfolding and aggregation and promotes intracellular trafficking and secretion, and it has been accepted that PBA was feasible for the treatment of urea-cycle disorders (Cao and Kaufman, 2014). Recently, PBA compounds demonstrated that ER stress might associate with the potential therapy of multiple diseases in clinical studies, including pancreatitis, alcoholic/non-alcoholic liver disease (Ji et al., 2011), and neuronal cell apoptosis (Mimori et al., 2012). Based on the foundation that ER stress is involved in the METH-induced damage of bEnd.3 cells, we used PBA *in vivo* to verify whether it could prevent or remedy METH-induced BBB disruption. Consequently, we observed that the METH-induced reduction of TJ protein expression followed by increased permeability was alleviated by PBA *in vivo*, suggesting that PBA could be a potential drug for METH-induced BBB disruption. Meanwhile, Shah and Kumar (2016) recently demonstrated that METH-induced programmed cell death in astrocytes was mediated by ER stress via ATF6, IRE1α, and PERK pathways. METH-induced ER stress in astrocytes could also be attributed to the therapeutic effect of PBA, since BBB was influenced by specific interactions between the brain’s endothelium and astrocytes underlying neurovascular units in both physiological and pathological conditions (Liebner et al., 2008; Artus et al., 2014). In the following studies, an *in vitro* BBB model will be established to illustrate the role of ER stress in different cell types of BBB and the interplay of these different cell types.

## Conclusion

**FIGURE 8:**
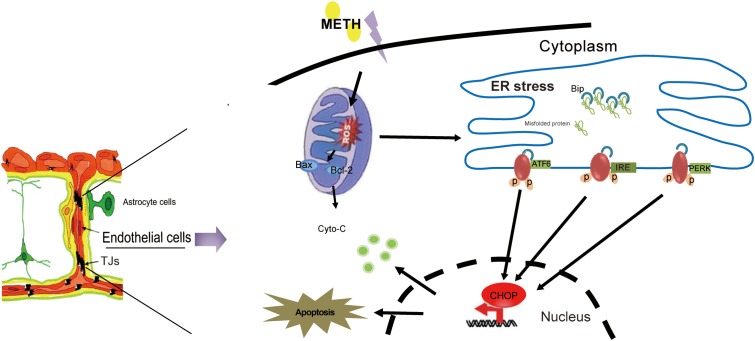
The model illustrates ER stress and mitochondrial dysfunction involving methamphetamine-induced brain endothelial cell dysfunction. METH induces the generation of ROS in brain endothelial cells, leading to prolonged ER stress. Prolonged ER stress induces the pro-apoptotic protein CHOP, which ultimately leads to increased cell apoptosis and decreased cell viability. Meanwhile, CHOP could further promote the mitochondria to release cytochrome c, accelerating cellular damage caused by ER stress.

In summary, our results showed that METH could disrupt BBB function by the induction of ER stress, OS and mitochondrial dysfunction in brain endothelial cells (**Figure 8**). Our findings provide novel insight into the mechanism of METH-induced brain endothelial injury. Therapies based on the inhibition of ER stress may be an effective and prospective treatment for METH-induced BBB disruption.

## Author Contributions

CM, BC, XQ, and DW conceived this work. XQ and DW wrote the main manuscript text. XQ and HG performed lab experiments. XQ and SL performed animal experiments. QS and YL analyzed and interpreted the data. GX and WZ cultivated the cells. XQ created the **Figures 1**–**7**; DW created the **Figure 8**. All authors read and commented on the manuscript.

## Conflict of Interest Statement

The authors declare that the research was conducted in the absence of any commercial or financial relationships that could be construed as a potential conflict of interest.
